# Olive Leaf as a Source of Antibacterial Compounds Active against Antibiotic-Resistant Strains of *Campylobacter jejuni* and *Campylobacter coli*

**DOI:** 10.3390/antibiotics12010026

**Published:** 2022-12-24

**Authors:** Jose Manuel Silvan, Esperanza Guerrero-Hurtado, Alba Gutierrez-Docio, Marin Prodanov, Adolfo J. Martinez-Rodriguez

**Affiliations:** 1 Microbiology and Food Biocatalysis Group (MICROBIO), Department of Biotechnology and Food Microbiology, Institute of Food Science Research (CIAL, CSIC-UAM), Autonomous University of Madrid, 28049 Madrid, Spain; 2 Department of Production and Characterization of Novel Foods, Institute of Food Science Research (CIAL, CSIC-UAM), Autonomous University of Madrid, 28049 Madrid, Spain

**Keywords:** *Campylobacter jejuni*, *Campylobacter coli*, antibiotic resistance, olive leaf extracts, antibacterial activity, hydroxytyrosol

## Abstract

*Campylobacter* spp. are the main cause of bacterial gastroenteritis worldwide, and broiler chicks are the main vector of transmission to humans. The high prevalence of *Campylobacter* in poultry meat and the increase of antibiotic resistant strains have raised the need to identify new antimicrobial agents. For this reason, the aim of the current study was to evaluate the antibacterial activity of two extracts of olive leaf against antibiotic-resistant *Campylobacter* strains (*C. jejuni* and *C. coli*) isolated from poultry food chain. The extracts of olive leaf (E1 and E2) were markedly different in their chemical compositions. While E1 was composed predominantly of highly hydrophilic compounds such as hydroxytyrosol and hydroxytyrosol glucosides (14,708 mg/100 g), E2 mainly contained moderately hydrophilic compounds, with oleuropein (20,471 mg/100 g) being prevalent. All *Campylobacter* strains exhibited similar antibiotic profiles, being resistant to ciprofloxacin and tetracycline. E1 showed strong antibacterial activity and reduced bacterial growth from 4.12 to 8.14 log CFU/mL, depending on the strain. Hydroxytyrosol was the main compound responsible, causing the inhibition of growth of *Campylobacter* strains at low concentrations (0.1–0.25 mg/mL). E2 demonstrated a lower antibacterial effect than E1, reducing growth from 0.52 to 2.49 log CFU/mL. The results of this study suggest that the optimization of the composition of olive-leaf extracts can provide improved treatment results against *Campylobacter* strains.

## 1. Introduction

*Campylobacter* spp., in particular *Campylobacter jejuni* (*C. jejuni*) and *Campylobacter coli* (*C. coli*), are the world’s leading cause of bacterial gastroenteritis, and campylobacteriosis is the most commonly reported zoonosis [1]. When *Campylobacter* enters the human body, it binds to the epithelial cells covering the gastrointestinal tract [2]. Later it invades these cells, inducing a severe inflammatory response that often results in diarrhea, fever, and cramps [3]. *Campylobacter* infection has also been associated with severe autoimmune diseases such as Guillain–Barré and Miller Fisher syndromes [4]. *Campylobacter* species are widely distributed in most warm-blooded animals [5], and chickens are a natural host for *Campylobacter.* It colonizes broiler chicks, which are the primary vector for transmitting this pathogen to humans [6]. Raw poultry meat is often contaminated with *Campylobacter*, since the bacterium can live in the intestines of healthy birds that may carry up to 10^9^ colony forming units (CFU) of *Campylobacter* per gram of cecal contents [7]. This high concentration of microorganisms allows *Campylobacter* to spread from chicken intestines into the meat during processing.

Acquired resistance to antibiotics in *Campylobacter*, as well as in other pathogens, represents a significant concern for the poultry industry and the consumer. The rise of *Campylobacter* strains resistant to antibiotics [8] has increased the need for new antimicrobials [9,10]. Among these new antimicrobials, certain plant extracts rich in different phenolic compounds have been shown to be effective against *Campylobacter* [11,12,13]. Although these plant extracts are generally less active than antibiotics, they can be effective against resistant strains [14,15]. For example, it has been reported that a 2 log CFU reduction in the number of campylobacters colonizing poultry can have a significant impact on consumer health, reducing incidence of human infection up to 30-fold [16,17,18]. In this regard, extracts prepared from olive oil industry by-products, such as olive mill wastewater and olive cake, have been shown to be effective as antibacterials against *C. jejuni* [14,19]. Supplementing broiler diets with different amounts of olive mill wastewater and olive cake resulted in a decrease in *Campylobacter* contamination [20]. However, the available information about the value of olive leaves as a source of antibacterial compounds for use against *Campylobacter* is scarce and contradictory. Olive leaves are the largest by-product obtained during olive tree pruning, olive harvesting, and cleaning. The amount of olive leaves accumulated annually by these methods may exceed 1 million tons [21]. Therefore, the development of processes that contribute to their revalorization is of particular importance. Some studies observed no antibacterial activity against *Campylobacter* in an extract of olive leaves with a high concentration of phenolic compounds [19]. However, other authors reported a high antibacterial activity in olive leaf extract against different *Campylobacter* strains, although they did not study the phenolic composition of the extract [22]. The leaf extracts obtained from different olive varieties that were effective against *Campylobacter* contained oleuropein and hydroxytyrosol as the main phenolic compounds [23]. These results suggest that the extract’s composition determines its antibacterial activity against *Campylobacter*. The present research analyzed antibacterial activity against different antibiotic-resistant strains of *C. jejuni* and *C. coli* isolated from the poultry food chain, and its relationship to the composition of two extracts of olive leaf, with the main purpose of identifying which components of the extract are linked to the antibacterial activity, in order to enhance their concentration and thus contribute to the formulation of more effective products against *Campylobacter*.

## 2. Results

### 2.1. Antibiotic Susceptibility of Campylobacter Strains

Antibiotic resistance profiles and minimal inhibitory concentration (MIC) values for *C. jejuni* and *C. coli* strains are shown in Table 1. Antibiotic susceptibility was similar for *C. jejuni* and *C. coli*. All *Campylobacter* strains isolated from the poultry food chain showed resistance to at least two of the six tested antibiotics. In contrast, the two reference strains (*C. jejuni* 700819 and *C. coli* 43478) did not show resistance to the tested antibiotics. This behavior is consistent with the rapid decline of bacterial drug-resistance in an antibiotic-free environment attained through the routine work with reference strains in the laboratory [24]. For this reason, when performing antibacterial activity assays it is important to use strains from recent isolates with few subcultures, as some collection strains or those with multiple laboratory passages may be particularly sensitive to antimicrobials.

All isolated strains were resistant to ciprofloxacin, which was a widely used antibiotic several years ago and nowadays encounters high levels of resistance in different countries [25,26,27]. It is known that the use of fluoroquinolones in poultry production is directly related to the emergence of resistant strains in humans [28]. A similar phenomenon was observed with tetracycline, which also displays high global resistance rates in humans as well as broilers [29,30]. No erythromycin-resistant strains were found, and this remains one of the most effective antibiotics against *Campylobacter* worldwide [8,31,32], although an increase of resistant strains has been reported in recent years, most notably for *C. coli* [33]. All strains were susceptible to gentamicin, which is also consistent with standard behavior against *Campylobacter* [8]. Amoxicillin–clavulanic acid, one of the most recommended therapies due to its successful results [34,35], was effective against all studied strains. However, the effectiveness was reduced when only amoxicillin was used, confirming that most *Campylobacter* strains can present resistance to this antibiotic, mediated by one or more of the three mechanisms of resistance to β-lactams described for *Campylobacter* (enzymatic inactivation by chromosomally-encoded β-lactamases, reduced uptake due to alterations in outer membrane porins, and efflux) [36].

Combined resistance to ciprofloxacin and erythromycin, which are both considered critically important for the treatment of campylobacteriosis [34], was not found. MIC values for antibiotics were generally greater in *C. coli* strains, suggesting a higher resistance of these strains to the antibiotics that were used. Multidrug resistance (MDR), which has been defined as a lack of susceptibility to at least one agent in three or more antibiotic categories [37], was observed in three strains of *C. jejuni* (JR1, JR2, and JP1). Low levels of *Campylobacter* MDR strains isolated from broilers have been reported in the European Union, being less than 1% for *C. jejuni* and close to 4% in the case of *C. coli* [8]. Complete susceptibility to the five antimicrobial classes was found only for the collection strains.

### 2.2. Chemical Characterization of Olive Leaf Extracts

**Figure 1:**
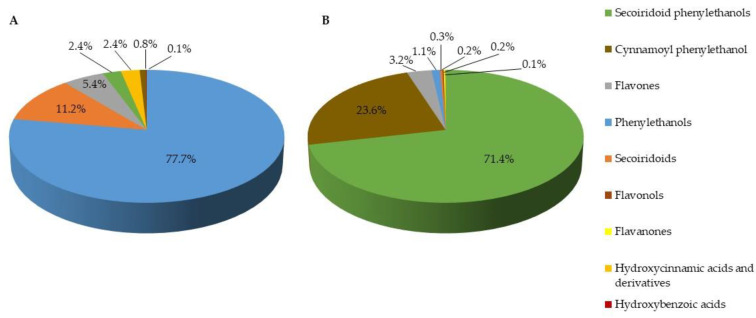
Phenolic and secoiridoid composition of olive-leaf extracts E1 (**A**) and E2 (**B**).

HPLC-PAD-MS analysis of E1 and E2 allowed the identification and quantification of twenty-eight known phenolic and secoiridoid compounds. Twenty-four were identified unambiguously and four (3,4-DHBA, 3,4-DHBA glucoside, and 3,4-DHPE glucosides) were identified tentatively (Table S1, Supplementary Material). Figure 1 shows the phenolic and secoiridoid composition of each extract. E1 and E2 were markedly different. While the predominant compounds in E1 were phenylethanols (78%) and secoiridoids (11%) (Figure 1A), the secoiridoid phenylethanols were the most abundant in E2 (72%), followed by cynnamoyl phenylethanols (24%) (Figure 1B). Other authors have reported that this variability in phenolic and secoiridoid composition among extracts of olive leaves is influenced by the extraction process and the type of processing, and by the storage stability of commercial products [38]. Factors such as olive variety, climate, location of the production area, and time of harvest also have significant influence on the phenolic and secoiridoid composition of the obtained final extracts [39].

Table 2 shows the phenolic and secoiridoid compounds identified in each of the extracts (E1 and E2). The total amount of compounds in E2 (29,155 mg/100 g) was higher than found in E1 (19,279 mg/100 g). Differences were also observed in the characteristics of the compounds present in each extract. E1 contained mainly highly hydrophilic compounds. Higher concentrations of hydroxytyrosol (13,743 mg/100 g), elenolic acid and associated glucosides (2164 mg/100 g), and hydroxycinnamic acids (465 mg/100 g) were detected. However, E2 contained mainly moderately hydrophilic compounds, the most abundant being oleuropein (20,471 mg/100 g) and verbascoside (6872 mg/100 g). Although oleuropein is the main component of olive fruits and leaves [40], it can be unstable and degrade to hydroxytyrosol under certain conditions, such as slightly acidic pH, high temperature, or exposure to light [41]. Luteolin, *trans*-4,5-DCQA, flavonols, and flavanones were not identified in extract E1, whereas phenylethanols, secoiridoids, and hydroxycinnamic and hydroxybenzoic acids, were detected in small amounts or were absent in extract E2.

### 2.3. Antibacterial Activity of Olive-Leaf Extracts against Campylobacter Strains

The antibacterial activity of the extracts of olive leaf against *C. jejuni* and *C. coli* strains is shown in Table 3. Antibacterial activity was directly related to the analytical composition of the extracts. E1 caused strong inhibition of growth in all strains, resulting in a decrease in bacterial growth between 4.12 and 8.14 log CFU/mL, depending on the analyzed strain. The extract was bactericidal (no growth detected) for two *C. jejuni* strains (JS1 and JR1) and for all *C. coli* strains. MIC values for E1 ranged between 1–2 mg/mL for *C. jejuni* strains and were about 10 times lower for *C. coli* strains (0.1–1.5 mg/mL). Although E2 significantly (*p* ≤ 0.05) reduced bacterial growth in most *Campylobacter* strains compared with the control (except *C. coli* CR2), this decrease was lower (from 0.52 to 2.49 log CFU/mL) than the reduction obtained with E1. MIC values for E2 (2 mg/mL) were also higher than those obtained for E1, for both *Campylobacter* species. The obtained results confirmed that it was not the total amount of phenolic and secoiridoid compounds present in the extracts (higher in the case of E2) that determined their antibacterial effectiveness against *Campylobacter* strains, but rather their composition in specific compounds.

Compared with E2, E1 showed a higher concentration of more hydrophilic compounds, with hydroxytyrosol and its glucoside forms as the major components (14,708 mg/g) (Table 2). It remains a matter of controversy whether the main antibacterial properties of olive oil and the extracts obtained from its by-products can be attributed to hydroxytyrosol. Some researchers have reported that hydroxytyrosol is a key component of the observed antibacterial effect against both Gram-positive and Gram-negative bacteria [42,43,44], while others suggest that the higher antibacterial capacity of olive oil and its derived by-products is better attributed to the dialdehydic form of decarboxymethyl elenolic acid or oleacein [45,46,47]. A study conducted with *E. coli* on the specific role of hydroxytyrosol as an antibacterial agent [48] concluded that the antibacterial effect of hydroxytyrosol was limited, and concentrations higher than 1 mg/mL are required to inhibit bacterial growth. However, no similar studies specifically in *Campylobacter* species have been reported, although some studies have suggested that hydroxytyrosol may play an important role in the antibacterial activity of olive oil and its by-products [14,23]. For this purpose, we evaluated the antibacterial effect of hydroxytyrosol (2 mg/mL) on two representative strains (*C. jejuni* JS1 and *C. coli* CR1) (Table 4). The results showed a bactericidal effect in both cases, similar to that obtained using E1 (Table 3). The MIC analysis in each case revealed that hydroxytyrosol at very low concentrations (0.1–0.25 mg/mL) was capable of inhibiting the growth of the *Campylobacter* strains.

Although other compounds such as elenolic acid 2-glucoside (oleoside 11-methyl ester) present in high concentrations in E1 have also been connected to antibacterial activity against *Campylobacter* [39], the essential role of hydroxytyrosol in E1 as an antibacterial agent against *Campylobacter* was evident. The final concentration of hydroxytyrosol in E1 for the antibacterial activity assay was 0.275 mg/mL, within the MIC interval found in experiments performed with pure hydroxytyrosol, indicating that in practice it should be possible to obtain commercial products with a high concentration of hydroxytyrosol and hence with putative antibacterial activity against *Campylobacter* strains. The antibacterial activity in E2 was much lower than in E1, confirming the limited antibacterial capacity of oleuropein, as described by others [39]. However, the high concentrations of oleuropein and verbascoside in the E2 extract make these a potential source of hydroxytyrosol by hydrolysis [42,49]. The standardization of hydroxytyrosol concentration in olive-leaf extracts would allow their optimization for use in the control of *Campylobacter*. In the case of poultry meat, these compounds could potentially be used at different stages of the food chain, from on-farm feed additives to packaging products [20,50].

## 3. Materials and Methods

### 3.1. Olive Leaf Extracts, Reagents, and Reference Substances

Extracts of olive leaf (E1 and E2) were provided by Pharmactive Biotech Products S.L. (Madrid, Spain). E1 was a water-soluble extract obtained by maceration of olive leaves in water and standardized in 4% elenolic acid and its derivatives (Isenolic^®^). Extract E2 was an alcohol-soluble extract obtained by maceration of olive leaves in a hydroalcoholic mixture and standardized in 20% oleuropein (Olivactive^®^). High-performance liquid chromatography (HPLC)-grade water was obtained using a Milli-Q purification system from Millipore Corp. (Bedford, MA, USA). HPLC-grade acetonitrile was purchased from Merck (Dramstadt, Germany), and acetic acid (99.8%) from Labbox Labware S.L. (Madrid, Spain). HPLC-grade pure reference substances *trans*-4,5-DCQA (*trans*-4,5-dicaffeoylquinic acid) (>95%), quercetin (>95%), 4-HPE-EA-glucoside (ligustroside) (>96.2%), and 3,4-DHPE-EA-glucoside (oleuropein) (>98%) were acquired from Merck. Elenolic acid (EA) (>98%) and luteolin (>95%) were purchased from Toronto Research Chemicals (Toronto, ON, Canada). The 3,4-DHBA (protocatechuic acid) (>90%), 4-HPE (tyrosol) (>95%), *trans*-4-HCA (*trans*-4-coumaric acid) (>98%), *trans*-3,4-DHCA (*trans*-caffeic acid) (>99%), *trans*-3-M,4-HCA (*trans*-ferulic acid) (>98%), quercetin 3-*O*-rhamnoside (quercitrin) (>93.3%), luteolin 3’,7-di-*O*-glucoside (>97%), eriodictyol-7-*O*-rutinoside (>98%), eriodictyol 7-*O*-glucoside (>98%), luteolin 7-*O*-glucoside (>98%), and 3,4-DHPE caffeoyl glucoside (verbascoside) (>95%) were obtained from Extrasynthese (Genay, France). EA 2-glucoside (oleoside 11-methyl ester) (>98%), EMA 2-glucoside (secoxyloganin) (>99%), 3,4-DHPE (hydroxytyrosol) (>90%), quercetin 3-*O*-glucoside (isoquercitrin) (>99%), apigenin 7-*O*-glucuronide (>90%), and luteolin 4′-methyl ether 7-*O*-glucoside (diosmin) (>90%) were purchased from PhytoLab GmbH & Co., KG (Vestenbergsgreuth, Germany). Apigenin 6,8-di-C-glucoside (>95%) was obtained from Glentham Life Sciences (Corsham, UK), and apigenin 7-*O*-rutinoside (isorhoifolin) (>99.9%) was obtained from Biosynth AG (Staad, Switzerland). The 3,4-DHPG (3,4-dihydroxyphenylglycol) (75%) was provided by Prof. Juan Fernández-Bolaños from Instituto de la Grasa (IG, CSIC) (Sevilla, Spain).

### 3.2. Chemical Characterization

Solutions of 20, 10, and 2 mg/mL of extracts E1 and E2 were prepared in water and methanol (*n* = 3), respectively, and were analyzed by reverse-phase HPLC (RP-HPLC), coupled to an ACE-3-C18-AR (200 mm × 4.6 mm, 3 μm particle size) column from Advanced Chromatography Technologies (Aberdeen, UK), a photodiode array detector (PAD), and mass spectrometry (MS) detector with electrospray ionization source (RP-HPLC-PAD-MS(ESI)) as described by Silvan et al. [51]. Samples of 3,4-DHBA, 3,4-DHPE, 4-HPE, 3,4-DHPE-EA-glucoside, 4-HPE-EA-glucoside, 3,4-DHPE caffeoyl glucoside, quercetin, quercetin 3-*O*-glucoside, quercetin 3-*O*-rhamnoside, apigenin 7-*O*-glucuronide, apigenin 6,8-di-C-glucoside, apigenin 7-*O*-rutinoside, luteolin, luteolin 3′,7-di-*O*-glucoside, luteolin 7-*O*-glucoside, luteolin 4′-*O*-methyl, 7-*O*-glucoside, eriodictyol 7-*O*-rutinoside, eriodictyol 7-*O*-glucoside, EA, EA 2-glucoside, EMA 2-glucoside, *trans*-3,4-DHCA, *trans*-4-HCA, *trans*-3-M,4-HCA, and *trans*-4,5-DCQA were identified unambiguously by co-elution and comparison with their retention times, order of elution, UV spectra, and the pseudomolecular and fragment ion masses of the corresponding pure reference substances, and quantified according to the calibration curves of each. The 3,4-DHBA, 3,4-DHBA glucoside, and 3,4-DHPE glucosides were identified tentatively using their corresponding retention time, order of elution, UV spectra, pseudomolecular and diagnostic fragment ion masses, and bibliographic data [52,53,54]. Then, 3,4-DHBA glucoside was quantified as equivalents of 3,4-DHBA, and 3,4-DHPE glucosides as equivalents of 3,4-DHPE. Results of quantification were expressed as mean value standard deviation (*n* = 3) for dry matter (mg/100 g).

### 3.3. Campylobacter Strains, Growth Media, and Culture Conditions

*Campylobacter jejuni* ATCC 700,819 and *Campylobacter coli* ATCC 43478, used as reference strains, were purchased from the American Type Culture Collection (ATCC) (Manassas, VA, USA). Eight *Campylobacter* strains (4 *C. jejuni* and 4 *C. coli* strains) were isolated from the poultry food chain (slaughterhouse, deboning, processing, and retail) following the procedure described elsewhere [55]. Identification at the species level was carried out by multiplex PCR [56] and confirmed through matrix-assisted laser desorption–ionization time-of-flight mass spectrometry (MALDI-TOF) (model Microflex LT) (Bruker Daltonics, Inc., Billerica, MA, USA) following the procedure described by Lapierre et al. [57]. The isolation sources and species of the strains used in this study are shown in Table 5. The strains were stored until use at −80 °C in Brucella Broth (BB) (Becton, Dickinson, & Co., Franklin Lakes, NJ, USA) with 20% glycerol. The agar plating medium consisted of Müeller–Hinton agar supplemented with 5% defibrinated sheep blood (MHB) (Becton, Dickinson, & Co.). The liquid growth medium for *Campylobacter* strains consisted of BB. The frozen strains were propagated by inoculation in MHB, followed by incubation under microaerophilic conditions (85% N_2_, 10% CO_2_, 5% O_2_) using a variable atmosphere incubator (VAIN) (MACS-VA500, Don Whitley Scientific, Shipley, UK) at 40 °C for 72 h. Isolated colonies were inoculated into 15 mL of BB and incubated while stirring at 150 rpm on an orbital shaker at 40 °C for 24 h in the VAIN under microaerophilic conditions. These bacterial inoculum cultures (~1 × 10^8^ CFU/mL) were used for the different experimental assays.

### 3.4. Determination of Antibiotic Susceptibility of Campylobacter spp. Strains

The antibiotic susceptibility of isolated *C. jejuni* and *C. coli* strains was determined for six of the most frequently used antibacterial agents, representing five different families (macrolides, tetracyclines, aminoglycosides, penicillins, and quinolones). Minimum inhibitory concentration (MIC) values for erythromycin (ERY), tetracycline (TET), gentamicin (GEN), amoxicillin (AMX), amoxicillin-clavulanic acid (AMC), and ciprofloxacin (CIP) were determined using E-test strips (BioMérieux, Madrid, Spain). This panel was selected in accordance with EUCAST guidelines and the recommendations of the European Centre for Disease Prevention and Control (ECDC) [58]. The control strains were *Campylobacter jejuni* ATCC 700819 and *Campylobacter coli* ATCC 43478. Bacterial inocula were prepared in BB, then 200 µL samples of this suspension were passed onto the surface of the MHB plates and streaked with a cotton swab. Antibiotic strips were placed on the surface of inoculated MHB plates. To test antibiotic susceptibility, the inoculated MHB plates were incubated in a microaerophilic incubator (VAIN) at 40 °C for 48 h before examination. MIC was measured according to the point where the ellipse growth intersected with the scale number on the E-test strip. The breakpoints were defined following European Committee on Antimicrobial Susceptibility Testing (EUCAST) guidelines (version 12.0) [59]. In cases of antibiotics that had no breakpoints available from EUCAST, data from La Société Française de Microbiologie [60] were used. Antibacterial concentration ranges applied in the tests and the breakpoints are summarized in Table 6.

### 3.5. Determination of Antibacterial Activity of Olive-Leaf Extracts against Campylobacter spp. Strains

The antibacterial activity of olive-leaf extracts (E1 and E2) against *Campylobacter* spp. was evaluated according to the protocol described by Silvan et al. [61]. Briefly, 1 mL samples of extracts were added into flasks containing 4 mL of BB. A final concentration of 2 mg/mL, in the range of interest for companies producing extracts [62], was used. Bacterial inoculum (50 µL of ~1 × 10^8^ CFU/mL) was then added to the flasks under aseptic conditions. Cultures were incubated with stirring (150 rpm) in a VAIN at 40 °C for 24 h. Controls were prepared using sterile water instead of extract. Decimal dilutions of cultures were prepared in saline solution (0.9% NaCl) after incubation, plated (20 L) onto fresh MHB agar, and incubated in a VAIN at 40 °C for 72 h. The numbers of CFU were assessed after incubation. The results of antibacterial activity were expressed as log CFU/mL (*n* = 3). MIC was determined following the procedure described above, and olive-leaf extracts (E1 and E2) were diluted in BB to obtain the desired final concentrations. MIC was defined as the lowest amount of extract that provoked a significant (*p* < 0.05) decrease in viability compared with the control growth after 24 h of treatment [12]. The dilution intervals for determination of MIC ranged from 0.1 to 2 mg/mL.

### 3.6. Verification of Antibacterial Activity Using Pure Hydroxytyrosol

Antibacterial properties of hydroxytyrosol, the major phenolic compound in E1, were investigated using a pure commercial standard (PhytoLab GmbH & Co. KG, Vestenbergsgreuth, Germany), following the procedures described in Section 3.5. All analyses were carried out using 2 mg/mL. MIC values for hydroxytyrosol were determined following the procedure described above, using pure commercial compound diluted in BB to obtain desired final concentrations ranging from 0.1 to 2 mg/mL.

### 3.7. Statistical Analysis

Results are reported as means ± SD. Significant differences among the data were estimated by applying analysis of variance (ANOVA) and *t* testing. Tukey’s least significant differences (LSD) test was employed to evaluate the significance of the analysis. Differences were considered significant at *p* < 0.05. All statistical tests were performed with IBM SPSS Statistics for Windows, Version 25.0 (IBM Corp., Armonk, NY, USA).

## 4. Conclusions

This work demonstrates that better treatment results and a reduction in the occurrence of discouraging responses can be obtained through the optimization of the composition of olive-leaf extracts for use as an antibacterial agent against *Campylobacter* strains. This could be achieved by enriching the levels of hydroxytyrosol in olive-leaf extracts, as this seems to be the most effective antibacterial compound against *Campylobacter*. The major phenolic compound in extracts of olive leaf is usually oleuropein, which is itself a source of hydroxytyrosol that can easily by obtained by hydrolysis. The ability of E1, rich in hydroxytyrosol, to inhibit *Campylobacter* growth demonstrates its potential to control the transmission of *Campylobacter* through the food chain, which should consequently contribute to reducing the incidence of campylobacteriosis without increasing the antibiotic resistance of this pathogen. Future work should focus on how different preparation methods and hydrolysis conditions of oleuropein-rich extracts can impact their antibacterial activity against *Campylobacter*, and the synergistic activity of these compounds with other phenolic groups present in the extract.

## Figures and Tables

**Table 1:** Antibiotic susceptibility profile and minimal inhibitory concentration (MIC) values for the *Campylobacter* spp. strains.

Specie	Strain	CIP	TET	ERY	GEN	AMC	AMX	Antibiotic Resistant Rate
*C. jejuni*	JS1	R (>32)	R (32)	S (0.5)	S (0.25)	S (0.5)	I (16)	2/6
	JR1	R (>32)	R (32)	S (0.5)	S (0.38)	S (0.5)	R (32)	3/6
	JR2	R (>32)	R (>256)	S (0.5)	S (0.19)	S (0.38)	R (>256)	3/6
	JP1	R (>32)	R (24)	S (0.38)	S (0.125)	S (0.25)	R (24)	3/6
	700819	I (0.032)	S (0.032)	S (0.25)	S (0.5)	S (0.19)	S (3)	0/6
*C. coli*	CR1	R (>32)	R (32)	S (0.75)	S (0.75)	S (1)	S (3)	2/6
	CP1	R (>32)	R (>256)	S (1)	S (0.5)	S (1)	I (6)	2/6
	CR2	R (>32)	R (3)	S (0.5)	S (0.5)	S (0.5)	S (2)	2/6
	CP2	R (>32)	R (>256)	S (2)	S (0.5)	S (0.75)	I (8)	2/6
	43478	I (0.016)	S (0.032)	S (0.125)	S (0.75)	S (2)	I (8)	0/6
Strain resistance rate		8/10	8/10	0/10	0/10	0/10	3/10	

S: susceptible; R: resistant; I: intermediate. MIC values are given in brackets.

**Table 2:** Quantification of main phenolic and secoiridoid compounds present in olive-leaf extracts (E1 and E2). Results are expressed as mean value ± standard deviation for dry matter (mg/100 g).

Compounds	Extract E1	Extract E2
***Phenylethanols***		
3,4-DHPE (Hydroxytyrosol) + 3,4-DHPE glucoside 1	13,743 ± 1659 *	182 ± 4 *
3,4-DHPE glucoside 2 + 3	965 ± 13 *	123 ± 1 *
4-HPE (Tyrosol)	250 ± 6 *	9.1 ± 0.1 *
3,4-DHPG	20.1 ± 0.4 *	9.4 ± 0.5 *
***Secoiridoids***		
EA 2-glucoside (Oleoside 11-methyl ester)	1352 ± 49 *	84.4 ± 4.6 *
EMA 2-glucoside (Secoxyloganin)	657 ± 158	ND
EA (Elenolic acid)	155 ± 14	ND
***Flavones***		
Luteolin 7-*O*-glucoside	655 ± 22 *	513 ± 46 *
Luteolin 4′-methyl ether 7-*O*-glucoside (Diosmin)	123 ± 11	111 ± 12
Apigenin 7-*O*-rutinoside (Isorhoifolin)	109 ± 1 *	122 ± 5 *
Apigenin 7-*O*-glucuronide	76.4 ± 7.1	64.0 ± 6.0
Luteolin 3′,7-di-*O*-glucoside	39.9 ± 1.4 *	69.6 ± 2.3 *
Apigenin 6,8-di-*C*-glucoside	39.3 ± 1.4 *	24.2 ± 0.2 *
Luteolin	ND	17.1 ± 1.2
***Secoiridoid phenylethanols***		
3,4-DHPE-EA glucoside (Oleuropein)	355 ± 57 *	20,471 ± 1061 *
4-HPE-EA-glucoside (Ligustroside)	99.3 ± 9.3 *	360 ± 16 *
***Hydroxycinnamic acids and derivatives***		
*trans*-4-HCA (*trans*-4-coumaric acid)	209 ± 45 *	1.2 ± 0.1 *
*trans*-3,4-DHCA (*trans*-caffeic acid)	140 ± 4 *	4.5 ± 0.1 *
*trans*-3-M,4-HCA (*trans*-ferulic acid)	116 ± 4 *	5.0 ± 0.5 *
*trans*-4,5-DCQA (*trans*-4,5-dicaffeoylquinic acid)	ND	16.5 ± 0.2
***Cynnamoyl phenylethanol***		
3,4-DHPE caffeoyl glucoside (Verbascoside)	161 ± 11 *	6872 ± 230 *
***Hydroxybenzoic acids***		
3,4-DHBA (Protocatechuic acid)	7.9 ± 0.2	ND
3,4-DHBA glucoside	6.4 ± 0.7	ND
***Flavonols***		
Quercetin 3-*O*-glucoside (Isoquercitrin)	ND	9.1 ± 0.6
Quercetin 3-rhamnoside (Quercitrin)	ND	10.8 ± 0.2
Quercetin	ND	31.4 ± 0.5
***Flavanones***		
Eriodictyol 7-*O*-rutinoside	ND	22.3 ± 3.1
Eriodictyol 7-*O*-glucoside	ND	22.8 ± 1.0
**Total phenolic and secoiridoid compounds**	**19,279**	**29,155**

ND: not detected; DHBA: dihydroxybenzoic acid; DHCA: dihydroxycinnamic acid; HCA: hydroxycinnamic acid; 3-M,4-HCA: 3-Methoxy-4-hydroxycinnamic acid; DCQA: dicaffeoylquinic acid; DHPG: 3,4-dihydroxyphenylglycol; DHPE: dihydroxyphenylethanol; HPE: hydroxyphenylethanol; EA: elenolic acid; EMA 2-glucoside: EA monoaldehyde isomer 2-glucoside. * Data marked with asterisk in the same row indicate significant difference between values according to *t* test (*p* ≤ 0.05).

**Table 3:** Antibacterial activity and minimal inhibitory concentration (MIC) of olive-leaf extracts (at 2 mg/mL) against *C. jejuni* and *C. coli* strains. The results are expressed in log CFU/mL ± standard deviation (SD) (*n* = 3).

Species	Strains	Control Growth	Extract E1			Extract E2		
			log CFU/mL	log Reduction	MIC (mg/mL)	log CFU/mL	log Reduction	MIC (mg/mL)
*C. jejuni*	JS1	9.62 ± 0.04 ^c^	<1.48 ^a^	>8.14	1.0	8.13 ± 0.09 ^b^	1.49	2.0
	JR1	8.63 ± 0.03 ^c^	<1.48 ^a^	>7.15	1.0	6.98 ± 0.09 ^b^	1.65	2.0
	JR2	8.50 ± 0.06 ^c^	2.67 ± 0.14 ^a^	5.83	2.0	6.86 ± 0.10 ^b^	1.64	2.0
	JP1	9.31 ± 0.06 ^c^	3.99 ± 0.04 ^a^	5.32	2.0	8.08 ± 0.04 ^b^	1.23	2.0
	700819	9.34 ± 0.05 ^c^	5.22 ± 0.04 ^a^	4.12	2.0	8.82 ± 0.01 ^b^	0.52	2.0
*C. coli*	CR1	8.64 ± 0.03 ^c^	<1.48 ^a^	>7.16	0.1	6.69 ± 0.02 ^b^	1.95	2.0
	CP1	9.32 ± 0.05 ^c^	<1.48 ^a^	>7.84	0.5	6.83 ± 0.06 ^b^	2.49	2.0
	CR2	9.47 ± 0.04 ^b^	<1.48 ^a^	>7.99	0.1	9.78 ± 0.03 ^b^	-	-
	CP2	9.16 ± 0.09 ^c^	<1.48 ^a^	>7.68	1.5	8.01 ± 0.07 ^b^	1.15	2.0
	43478	8.47 ± 0.05 ^c^	<1.48 ^a^	>6.99	0.1	6.22 ± 0.05 ^b^	2.25	2.0

Colony-forming unit (CFU) detection limit was 1.48 log CFU/mL (30 CFU per plate). ^a,b,c^ Log CFU/mL values in the same row marked with different letters indicate significant differences according to ANOVA post hoc LSD Tukey test (*p* ≤ 0.05).

**Table 4:** Antibacterial activity and minimal inhibitory concentration (MIC) of hydroxytyrosol (2 mg/mL) against *C. jejuni* JS1 and *C. coli* CR1 strains. The results are expressed in log CFU/mL ± standard deviation (SD) (*n* = 3).

Strains	Control Growth	Hydroxytyrosol	log Reduction	MIC (mg/mL)
*C. jejuni* JS1	9.49 ± 0.05	<1.48	>9.49	0.25
*C. coli* CR1	8.76 ± 0.03	<1.48	>8.76	0.1

Colony-forming unit (CFU) detection limit was 1.48 log CFU/mL (30 CFU per plate).

**Table 5:** Summary of *Campylobacter* spp. strains isolated from different stages of the chicken food chain and collection strains used in the present study.

Strain Designation	Specie	Isolation Source
JS1	*C. jejuni*	Slaughterhouse
JR1	*C. jejuni*	Chicken breast retail
JR2	*C. jejuni*	Chicken drumstick retail
JP1	*C. jejuni*	Carcass in production chain
700819	*C. jejuni*	Reference strain *
CR1	*C. coli*	Chicken drumstick retail
CP1	*C. coli*	Machine in production chain
CR2	*C. coli*	Hamburger retail
CP2	*C. coli*	Carcass in production chain
43478	*C. coli*	Reference strain *

All strains used are part of the MICROBIO group collection. * Bacterial strain was obtained from the American Type Culture Collection (ATCC).

**Table 6:** Overview of the tested antibacterial agents, antibacterial concentration ranges, and corresponding breakpoints for *C. jejuni* and *C. coli*.

Antibiotic Group	Antibiotic Agent	Concentration Range (µg/mL)	Breakpoint (S/R) MIC (µg/mL)	
			*C. jejuni*	*C. coli*
Macrolides	Erythromycin (ERY)	0.016–256	≤4/>4 ^1^	≤8/>8 ^1^
Tetracyclines	Tetracycline (TET)	0.016–256	≤2/>2 ^1^	≤2/>2 ^1^
Aminoglycosides	Gentamicin (GEN)	0.016–256	≤2/>2 ^2^	≤2/>2 ^2^
Penicillins	Amoxicillin (AMX)	0.016–256	≤4/>16 ^2^	≤4/>16 ^2^
	Amoxicillin/Clavulanic acid (AMC)	0.016–256	≤4/>16 ^2^	≤4/>16 ^2^
Fluoroquinolones	Ciprofloxacin (CIP)	0.002–32	≤0.001/>0.5 ^1^	≤0.001/>0.5 ^1^

S = susceptible; R = resistant. ^1^ Breakpoints established by EUCAST (EUCAST guideline version 12.0, January 2022). ^2^ Breakpoints established by La Société Française de Microbiologie (SFM, 2022).

## Data Availability

The data presented in this study are available in this manuscript.

## Supplementary Materials

The following supporting information can be downloaded at: https://www.mdpi.com/article/10.3390/antibiotics12010026/s1, Table S1: Ultraviolet absorption, mass spectrometric data of main phenolic and secoiridoid compounds present in olive-leaf extracts (E1 and E2).
