# Supplementary material for: Olive Leaf as a Source of Antibacterial Compounds Active against Antibiotic-Resistant Strains of Campylobacter jejuni and Campylobacter coli
Source: Antibiotics (Basel). 2022 Dec 24;12(1):26. doi: 10.3390/antibiotics12010026 (PMC9854969; doi:10.3390/antibiotics12010026)
Supplement: Supplementary file 1 [file antibiotics-12-00026-s001.zip › antibiotics-2087578-supplementary.pdf]

Supplementary Table (S1). Ultraviolet absorption, mass spectrometric data of main phenolic and secoiridoid compounds present in olive-leaf extracts (E1 and E2).

Compounds	Abs_{max} (nm)	$[M + H]^+$ (*m*/*z*)	[M – H] (*m*/*z*)	Productions (-) (*m*/*z*)
Phenylethanols and glycosides				
3,4-DHPE (Hydroxytyrosol) + 3,4-DHPE glucoside 1	234/278		153.1<br>315.1	305.2† ; 123.2<br>153.1 ; 123.2
3,4-DHPE glucoside 2 + 3	230/278		315.0	153.1
4-HPE (Tyrosol)	232/275	161.2 $[M+Na]^+$<br>139.0		
3,4-DHPG	232/278		169.0	
Secoiridoids				
EA 2-glucoside (Oleoside 11-methyl ester)	238		403.1	807.2† ; 223.1 ; 179.0 ; 119.1 ; 112.9
EMA 2-glucoside (Secoxyloganin)	237		403.0	807.2† ; 223.1 ; 179.1 ; 121.1 ; 119.2 ; 113.1
EA (Elenolic acid)	239		241.1	165.1 ; 139.1 ; 127.1 ; 121.1 ; 111.2 ; 101.0
Flavones				
Luteolin 7-*O*-glucoside	254/268sh/348	449.1	447.1	895.0†
Luteolin 4′-methyl ether 7-*O*-glucoside (Diosmin)	252/266/347	609.2	606.9	461.0 ; 299.1
Apigenin 7-*O*-rutinoside (Isorhoifolin)	266/341	579.1	576.8	
Apigenin 7-*O*-glucuronide	266/338		445.1	
Luteolin 3′,7-di-*O*-glucoside	267/342		609.1	
Apigenin 6,8-di-C-glucoside	254/265sh/348	595.2		
Luteolin	253/265/348	287.0	285.1	284.1
Secoiridoid phenylethanols				
3,4-DHPE-EA glucoside (Oleuropein)	234/280	563.2 $[M+Na]^+$	539.2	
4-HPE-EA-glucoside (Ligustroside)	234/280		523.0	
Hydroxycinnamic acids and derivatives				
trans-4-HCA (*trans*-4-coumaric acid)	233/296sh/308		163.0	145.0 ; 123.1 ; 119.1
trans-3,4-DHCA (*trans*-caffeic acid)	234/296sh/322	181.0	179.0	135.1
trans-3-M,4-HCA (*trans*-ferulic acid)	236/295sh/322		193.1	178.1 ; 149.0
trans-4,5-DCQA (*trans*-4,5-dicaffeoylquinic acid)	235/300sh/326	517.1		
Cynnamoyl phenylethanol glycosides				
3,4-DHPE caffeoyl glucoside (Verbascoside)	234/285sh/330	647.2 $[M+Na]^+$	623.2	
Hydroxybenzoic acids and glycosides				
3,4-DHBA (Protocatechuic acid)	260/294		153.0	123.1 ; 109.0
3,4-DHBA glucoside	253/293		314.9	153.0 ; 136.9
Flavonols				
Quercetin 3-*O*-glucoside (Isoquercitrin)	233/281/322	465.1	463.1	
Quercetin 3-rhamnoside (Quercitrin)	253/295sh/342	449.1	447.1	
Quercetin	254/300sh/370	303.0		
Flavanones				
Eriodictyol 7-*O*-rutinoside	233/283/328		594.6	449.1 ; 286.9 ; 151.3
Eriodictyol 7-*O*-glucoside	232/277/340	451.1	449.1	

sh: peak shoulder; DHBA: dihydroxybenzoic acid; DHCA: dihydroxycinnamic acid; HCA: hydroxycinnamic acid; 3-M,4-HCA: 3-Methoxy-4-hydroxycinnamic acid; DCQA: dicaffeoylquinic acid; DHPG: 3,4-dihydroxyphenylglycol; DHPE: dihydroxyphenylethanol; HPE: hydroxyphenylethanol; EA: Elenolic acid; EMA 2-glucoside: EA monoaldehyde isomer 2-glucoside. † Double-charged ion $[M-2H]^{2-}$.
